# 582 Predictors of Care Discontinuity in a National Sample of Burn Patients

**DOI:** 10.1093/jbcr/irae036.216

**Published:** 2024-04-17

**Authors:** Manuel Castillo-Angeles, Lauren Shepler, Caitlin M Orton, Colleen M Ryan, Jeffrey C Schneider, Anupama Mehta

**Affiliations:** Brigham and Women's Hospital, Brookline, MA; Spaulding Rehabilitation Hospital, Harvard Medical School, Boston, MA; UW Medicine Regional Burn Center, Harborview Medical Center, Seattle, WA; Massachusetts General Hospital/Shriners Children's, Boston, MA; Brigham and Women's Hospital, Boston, MA; Brigham and Women's Hospital, Brookline, MA; Spaulding Rehabilitation Hospital, Harvard Medical School, Boston, MA; UW Medicine Regional Burn Center, Harborview Medical Center, Seattle, WA; Massachusetts General Hospital/Shriners Children's, Boston, MA; Brigham and Women's Hospital, Boston, MA; Brigham and Women's Hospital, Brookline, MA; Spaulding Rehabilitation Hospital, Harvard Medical School, Boston, MA; UW Medicine Regional Burn Center, Harborview Medical Center, Seattle, WA; Massachusetts General Hospital/Shriners Children's, Boston, MA; Brigham and Women's Hospital, Boston, MA; Brigham and Women's Hospital, Brookline, MA; Spaulding Rehabilitation Hospital, Harvard Medical School, Boston, MA; UW Medicine Regional Burn Center, Harborview Medical Center, Seattle, WA; Massachusetts General Hospital/Shriners Children's, Boston, MA; Brigham and Women's Hospital, Boston, MA; Brigham and Women's Hospital, Brookline, MA; Spaulding Rehabilitation Hospital, Harvard Medical School, Boston, MA; UW Medicine Regional Burn Center, Harborview Medical Center, Seattle, WA; Massachusetts General Hospital/Shriners Children's, Boston, MA; Brigham and Women's Hospital, Boston, MA; Brigham and Women's Hospital, Brookline, MA; Spaulding Rehabilitation Hospital, Harvard Medical School, Boston, MA; UW Medicine Regional Burn Center, Harborview Medical Center, Seattle, WA; Massachusetts General Hospital/Shriners Children's, Boston, MA; Brigham and Women's Hospital, Boston, MA

## Abstract

**Introduction:**

Continuity of care has become a widely accepted metric of quality of care. It has been shown that care discontinuity is associated with worse outcomes in the surgical and burn populations. However, there is a scarcity of data on factors leading to this discontinuity. The aim of this study was to determine predictors of care discontinuity in a national sample of burn patients.

**Methods:**

This was a retrospective study using a multicenter longitudinal database from 1996 to 2021. All adult burn patients from participating sites were included. The main outcome was care discontinuity. This was defined by having therapy or burn-related surgeries at non-index (different hospital than the one for the original admission). Multivariable logistic regression was used to examine the association between multiple demographic and clinical factors and care discontinuity.

**Results:**

A total of 4,167 burn patients were included. Mean age was 43.36 (SD 15.85) years, 73% were male and 70% were white. 505 (12.1%) burn patients reported having therapy or burn-related surgeries at a non-index hospital. After adjusted analysis, we identified several predictors of care discontinuity in the burn population. Older age (Odds Ratio [OR] 1.02, 95% Confidence Interval [CI] 1.01 – 1.03), female sex (OR 1.28, 95% CI 1.01 – 1.62), Total Body Surface Area burned (OR 1.01, 95% CI 1.01 – 1.02) and number of trips to the operating room (OR 1.26, 95% CI 1.19 – 1.32) were associated with higher odds of being readmitted to a non-index hospital. However, racial minority (OR 0.65, 95% CI 0.49 – 0.88) and a longer hospital stay (OR 0.99, 95% CI 0.98 – 0.99) were associated with a lower risk of care discontinuity.

**Conclusions:**

Care discontinuity was not that common in the burn population, and it is mainly driven by patient demographic and burn-specific clinical characteristics. Further research should focus on developing strategies targeted to address these disparities in order to improve continuity of care in the burn population.

**Applicability of Research to Practice:**

Understanding the predictors of care discontinuity in the burn population may help clinicians develop strategies to address these disparities, decrease care fragmentation and subsequently have better outcomes.

